# MAML1 Acts Cooperatively with EGR1 to Activate EGR1-Regulated Promoters: Implications for Nephrogenesis and the Development of Renal Cancer

**DOI:** 10.1371/journal.pone.0046001

**Published:** 2012-09-27

**Authors:** Magnus L. Hansson, Stefanie Behmer, Rebecca Ceder, Sadollah Mohammadi, Giulio Preta, Roland C. Grafström, Bengt Fadeel, Annika E. Wallberg

**Affiliations:** Division of Molecular Toxicology, Institute of Environmental Medicine, Karolinska Institutet, Stockholm, Sweden; Pontificia Universidad Catolica de Chile, Chile

## Abstract

Mastermind-like 1 (MAML1) is a transcriptional coregulator of activators in various signaling pathways, such as Notch, p53, myocyte enhancer factor 2C (MEF2C) and beta-catenin. In earlier studies, we demonstrated that MAML1 enhanced p300 acetyltransferase activity, which increased the acetylation of Notch by p300. In this study, we show that MAML1 strongly induced acetylation of the transcription factor early growth response-1 (EGR1) by p300, and increased EGR1 protein expression in embryonic kidney cells. *EGR1* mRNA transcripts were also upregulated in the presence of MAML1. We show that MAML1 physically interacted with, and acted cooperatively with EGR1 to increase transcriptional activity of the EGR1 and p300 promoters, which both contain EGR1 binding sites. Bioinformatics assessment revealed a correlation between *p300*, *EGR1* and *MAML1* copy number and mRNA alterations in renal clear cell carcinoma and *p300*, *EGR1* and *MAML1* gene alterations were associated with increased overall survival. Our findings suggest MAML1 may be a component of the transcriptional networks which regulate EGR1 target genes during nephrogenesis and could also have implications for the development of renal cell carcinoma.

## Introduction

Mastermind-like 1 (MAML1) is the human homolog of *Drosophila* Mastermind, a neurogenic gene genetically linked to Notch function [1], [2]. The Notch signaling pathway plays an important role in many developmental processes by influencing cellular proliferation, differentiation and apoptosis. In the nucleus, the Notch intracellular domain (Notch ICD) associates with the transcription factor CSL (also known as RBP-Jk or CBF1 in vertebrates, Suppressor of Hairless in *Drosophila* and Lag-1 in *C. elegans*) when it is bound to DNA, and coactivators such as PCAF [3], GCN5 [3], p300 [4] and MAML1 [1], [2] are recruited to activate the expression of Notch target genes. Recently, MAML1 has been shown to function as a coactivator for transcription factors involved in a variety of Notch-independent signaling pathways, including myocyte enhancer factor 2C (MEF2C) [5], p53 [6] and beta-catenin [7], and the N-terminus of MAML1 is crucial for these interactions. These findings suggest that MAML1 functions as a coactivator in diverse cellular processes and may be a mediator of crosstalk between different signaling pathways. MAML1 may also affect various signaling pathways by interacting with p300, a commonly used coactivator. We and other researchers have previously reported that MAML1 mediates Notch ICD-mediated transcription in vivo, and can also form chromatin templates in vitro, by recruiting p300 to a DNA-CSL-Notch complex [8], [9], [10]. We recently reported that MAML1 enhances p300 autoacetylation, histone acetyltransferase (HAT) activity and coactivator function [11]. We also found that p300 acetylates the Notch1 ICD in cell culture assays and *in vitro*, and that regions located within the Notch C-terminus are essential for Notch acetylation. MAML1 and CSL, components of the Notch transcription complex, strongly enhance Notch acetylation and we suggested that MAML1 increases Notch acetylation by potentiating p300 autoacetylation [12]. p300 can also acetylate MAML1, although the effect of this posttranslational modification on the function of MAML1 is currently unknown. MAML1 is a phosphoprotein, and we previously identified GSK3ß as one kinase responsible for phosphorylation of MAML1 *in vitro* [13]. The active form of GSK3ß directly interacts with the N-terminus of MAML1 to inhibit MAML1 transcriptional activity [13]. In addition, the MAML1 N-terminus is subject to SUMOylation, which also represses the transcriptional activity of MAML1 [14].

The transcription factor early growth response-1 (EGR1) is expressed in response to various extracellular signals, such as growth factors, cytokines, irradiation, and various kinds of stress. In many normal cells, growth factor stimulation rapidly induces expression of EGR1, which subsequently leads to the activation of downstream growth pathways [15]. However, EGR1 can also suppress growth when overexpressed in transformed cells in several experimental systems [16]. The cellular response to EGR1 with respect to apoptosis varies: EGR1 can induce apoptosis by stimulating either p53 or PTEN; however, EGR1 can also promote survival in certain cell types by counteracting p53-dependent apoptosis [17]. EGR1 plays an important role as a tumor suppressor in breast, brain and lung cancer, as EGR1 is poorly expressed in these tissues and acts as a suppressor of growth and transformation when overexpressed [16]. In contrast, expression of EGR1 appears to be maintained in a high proportion of prostate and kidney cancers, where it promotes growth. EGR1 is absent or expressed at low levels in normal prostate tissues; whereas the EGR1 promoter is regulated by a positive feedback loop between EGR1 and growth factors in prostate cancer cells, which results in constitutive growth. Prostate cancer tissues also express high levels of p300, which leads to constitutively high levels of stable acetylated EGR1 protein, which is an important component in the transformation and progression of this disease [18]. The levels of EGR1 increase with the degree of malignancy in prostate cancer, as indicated by the Gleason tumor score [15]. The ability to reduce EGR1 expression may provide an efficient clinical treatment for prostate cancer, as downregulation of EGR1 may reduce growth factor induction and positive feedback to the EGF1 promoter.

EGR1 has been implicated as an important factor in nephrogenesis and the development of renal cancer. During embryogenesis, EGR1 is expressed in almost all proliferating cells including metanephric blastems; however, it is normally downregulated during renal development [19]. Expression of EGR1 is also elevated in many Wilms' tumors compared to normal kidney tissues. Wilms' tumor is a pediatric malignancy of the kidney, which is often of embryonic origin [20]. Overexpression of EGR1 has been reported to increase proliferation, enhance tumor growth and antagonize the effects of the tumor suppressor Wilms tumor 1 (WT1) in baby rat kidney cells [21]. Renal cell carcinoma (RCC) is the most common kidney cancer in adults, accounting for 80–85% of primary kidney malignances. Clear cell (conventional) RCC and renal papillary cell carcinoma are the most and second-most frequent types of RCC, respectively. Strefford and colleagues reported that chromosome 5 was overrepresented and rearranged in a significant proportion of 19 renal cell carcinoma cell lines, with the genes encoding *EGR1* (5q31.1) and *CSF1R* (Colony-stimulating factor 1 receptor) (5q33–q35) most frequently altered on chromosome 5, further supporting a role for EGR1 in renal cancer [22]. In a previous study, we reported that MAML1 increased p300 autoacetylation and p300 acetylation activity in embryonic kidney (HEK293) cells [11]. The objective of the present study was to investigate whether MAML1 and p300 regulate the levels of EGR1 in kidney cells.

## Materials and Methods

### Plasmids

pcDNA3.1-FLAG-EGR1 and pGL3-p300 were purchased from Addgene (Cambridge, MA, USA). The plasmid 4xEBS1-luc was a kind gift from Dr. G. Thiel (University of Saarland Medical Center, Homburg, Germany) and pGl3-EGR1 was generously provided by Dr. T.E. Eling (National Institute of Environmental Health Sciences, NC, USA). pcDNA3.1-FLAG-MAML1 (1–1016), (1–625) and cmv-p300-HA have been described previously [11]. The cDNA encoding MAML1 (1–127) and (75–1016) were amplified by PCR and subcloned into the expression vector FLAG-pcDNA3.1.

### Cell lines

The MAML1 cell lines were constructed by transfecting human embryonic kidney (HEK)-293 cells with the vectors pcDNA3.1-FLAG-MAML1 or pcDNA3.1-MAML1 and stable clones were selected with geneticin [11].

### siRNA transfection

HEK-293 cells were transiently transfected with 100 nM MAML1 siRNA (predesigned MAML1 SMARTpool set of 4 siRNAs; Dharmacon) or control siRNA using DharmaFECT 1 siRNA reagents (Dharmacon, Lafayette Colorado, USA), and cultured in the presence or absence of 50 ng/ml TPA (12-O-Tetradecanoylphorbol-13-Acetate) (Cell Signaling Technology, Boston, MA, USA).

### Reporter gene assay

HEK-293 cells in 24-well plates were transiently transfected using TransIT-LT1 reagent (Mirus, Madison, WI, USA) with 200 ng reporter plasmid DNA and 150 ng EGR1 or MAML1 expression vector DNA, as indicated in the figures. The cells were lysed in Reporter Lysis buffer (Promega, Madison, WI, USA) after 24 h and luciferase activity was measured using LucySoft3. The data are reported as the mean values of at least three independent experiments.

### Real-time PCR

Total RNA was purified using the RNeasy mini kit (Qiagen, Hilden, Germany) according to the manufacturer's protocol and cDNA was synthesized using the Superscript III first-strand synthesis system (Invitrogen, Carlsbad, CA, USA). Real-time PCR was performed using Power SYBR Green master mix (Applied Biosystems, Foster City, CA, USA) on an Applied Biosystems 7500 Sequence detector using the following primers sets: *EGR1* forward, 5′-TACGAGCACCTGACCGCAG-3′;

reverse, 5′-CACCAGCACCTTCTCGTTGTT-3′;

18S rRNA forward, 5′-CCTGCGGCTTTAATTTGACTCA-3′;

reverse, 5′-AGCTATCAATCTGTCAATCCTGTC-3′.

*EGR1* mRNA expression was normalized to 18S rRNA in each sample.

### *In vivo* acetylation assay

HEK-293 cells were transiently transfected with pcDNA3.1-FLAG-EGR1, pcDNA3.1-MAML1 and cmv-p300-HA using TransIT-LT1 transfection reagent (Mirus, Madison, WI, USA). After 24 h, the cells were treated with 10 mM sodium butyrate. The cells were harvested 40 h after transfection, lysed in lysis buffer (50 mM Tris-HCl pH 8, 150 mM NaCl, 1% Triton X-100 and complete EDTA-free protease inhibitor Pierce). Immunoprecipitation of FLAG-EGR1 was performed using M2-agarose that recognizes the FLAG epitope. The input and immunoprecipitation (IP) samples were analyzed by Western blotting using the following antibodies: EGR1 (Santa Cruz Biotechnology, CA, USA), the acetylated lysines in EGR1 (Cell Signaling Technology, Boston, MA, USA), p300 (Santa Cruz Biotechnology, CA, USA), MAML1 (Bethyl Laboratories, Cambridge, United Kingdom; Millipore, Billerica, MA, USA) and acetylated p300 lysine 1499 (Cell Signaling Technology, Boston, MA, USA).

### Co-immunoprecipitation

Whole-cell lysates from HEK-293 cells were pre-cleared and endogenous MAML1 was immunoprecipitated using an MAML1 antibody (Santa Cruz Biotechnology, CA, USA). The input and IP samples were analyzed by Western blotting using the following antibodies: MAML1 (Bethyl laboratories, Cambridge, United Kingdom) and EGR1 (Santa Cruz Biotechnology, CA, USA).

### Immunostaining

HCT116 cells were transfected with pcDNA3-FLAG-EGR1 and pcDNA3.1-MAML1 using TransIT-LT1 transfection reagent (Mirus, Madison, WI, USA), incubated for 24 h, washed with PBS, fixed with 4% paraformaldehyde, and permeabilized with 0.5% Triton X-100 (Sigma-Aldrich, St. Louis, Missouri, USA). The cells were immunostained using FLAG (Sigma-Aldrich, St. Louis, Missouri, USA) and MAML1 (Millipore, Billerica, MA, USA) antibodies, and analyzed using a Leica confocal microscopy (Leica Microsystems, Wetzler, Germany).

### GST pull-down assay

HEK293 cells were transfected with pcDNA3.1-FLAG-EGR1 using TransIT-LT1 transfection reagent, and cultured for 2 h with 50 ng/ml TPA 24 h post-transfection. Whole cell lysates were prepared and 100 µl aliquots of protein extract in lysis buffer (50 mM Tris-HCl pH 8, 150 mM NaCl, 1% Triton X-100, 1 mM DTT and 1× complete EDTA-free protease inhibitor cocktail) were incubated with GST proteins (prepared as described in [14]) bound to Glutathione Sepharose 4B beads (GE Healthcare, Little Chalford, United Kingdom) in BC-150 buffer (50 mM Tris-HCl pH 7.5, 150 mM KCl, 10% glycerol) overnight at 4°C. After washing with BC-150 buffer, the bound proteins were eluted in SDS-PAGE loading buffer (1 M Tris-HCl pH 6.8, 50% glycerol, 10% SDS, 1% bromophenol blue, 1 mM DTT), separated using 7.5% SDS-polyacrylamide gels, transferred to PVDF membranes (GE Healthcare, Little Chalford, United Kingdom) and incubated with an antibody recognizing EGR1 (Santa Cruz Biotechnology, CA, USA).

### Protein stability assay

HEK-293 FLAG-MAML1 and HEK-293 control cells were cultured in 24-well plates and treated with 50 ng/ml TPA for 2 h and 50 µg/ml cyclohexamide (Sigma-Aldrich, St. Louis, Missouri, USA) for 1 h. The cells were harvested in lysis buffer (50 mM Tris-HCl pH 8, 150 mM NaCl, 1% Triton X-100 and complete EDTA-free protease inhibitor cocktail), the proteins were separated using SDS-PAGE and subjected to immunoblotting using an antibody recognizing EGR1 (Santa Cruz Biotechnology, CA, USA). The intensities of the bands were quantified with Image J (rsb.info.nih.gov/ij/index.html).

### Bioinformatic analysis of *MAML1, EGR1* and *p300*

Expression of *MAML1*, *EGR1* and *p300* were analyzed in tumor samples from 20 different cancer studies using the web-based cBio Cancer Genomics Portal (http://www.cbioportal.org/, last accessed 07/26/12) including genomics data, e.g., DNA copy number data from 5134 samples (array-comparative genomic hybridization) and mRNA expression data from 2430 samples (RNA-sequencing). Putative copy number alterations such as amplifications and homozygous deletions were derived within the portal using the “genomic identification of significant targets in cancer” algorithm [23]. The mRNA expression changes were considered significant if samples displayed a Z-score ≥2 compared to the reference population (diploid tumors or normal adjacent tissues if applicable). The association of altered gene expression with overall survival was assessed in selected cancer studies using Kaplan-Meier analysis. The patient samples were divided into two groups based on “gene set altered” or “gene set not altered” using the selected genomics data, and significant survival differences between the groups were determined using the log-rank test; *p*-values <0.05 were considered statistically significant.

## Results and Discussion

### MAML1 regulates EGR1 mRNA and protein expression

**Figure 1:**
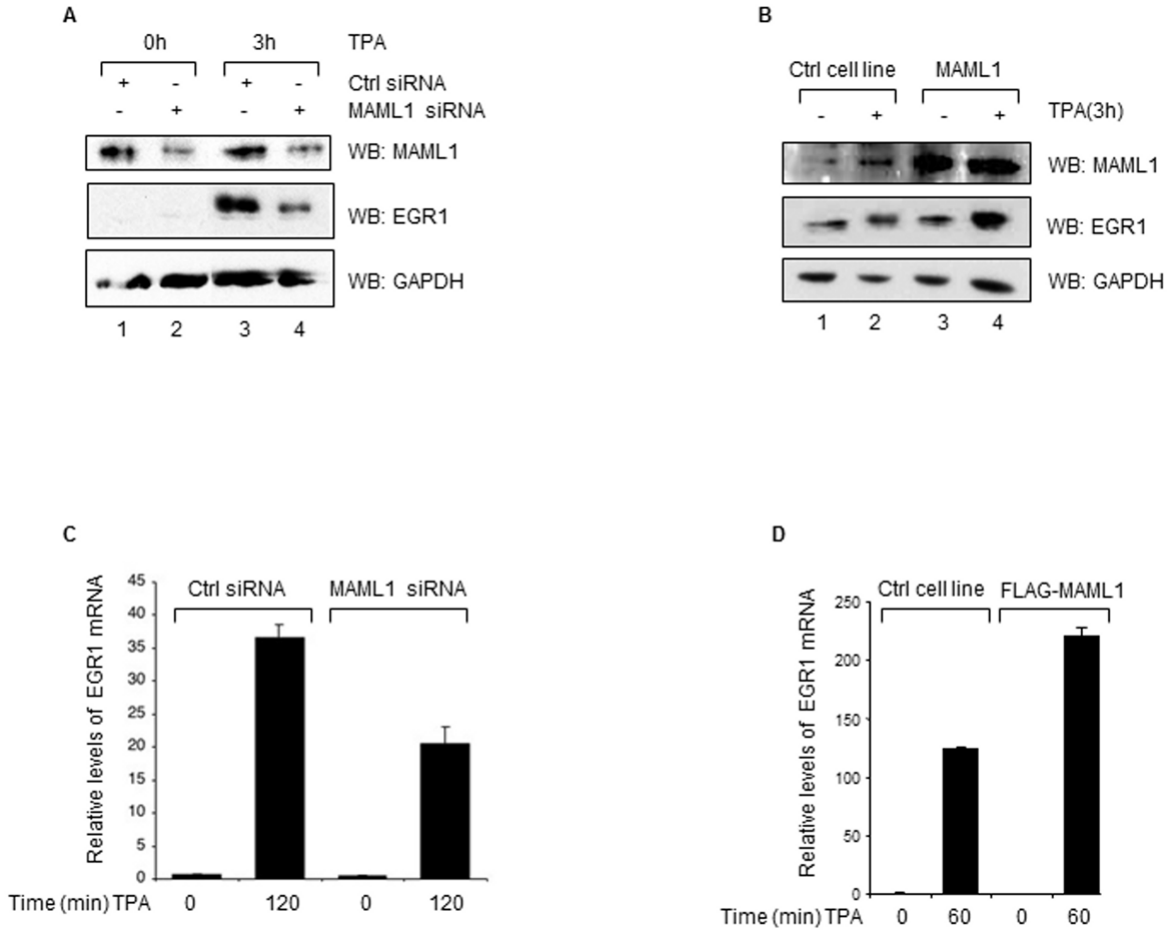
MAML1 regulates EGR1 mRNA and protein expression. (**A**) HEK-293 cells were transfected with *MAML1* siRNA or control (Ctrl) siRNA, and cultured in the presence of TPA to induce expression of EGR1. Proteins were detected by immunoblotting using antibodies recognizing MAML1, EGR1 and GAPDH. (**B**) Whole cell extracts were prepared from cells expressing MAML1 and control HEK-293 cells cultured with TPA. Proteins were detected by immunoblotting using the indicated antibodies. (**C**) HEK-293 cells were transfected with *MAML1* siRNA or control siRNA, cultured with TPA; then *EGR1* mRNA expression was analyzed by real-time PCR. (**D**) HEK-293 control cells and HEK-293 cells stably expressing FLAG-MAML1 were cultured with TPA; then *EGR1* mRNA expression was analyzed by real time PCR. The data is presented as the mean ± standard error of the mean (SEM) and expressed relative to *EGR1* levels in control HEK-293 cells cultured in the absence of TPA.

EGR1 protein stability has been reported to be stabilized by p300 acetylation, which results in the transactivation of survival genes [18]. As we previously found that MAML1 increases p300 autoacetylation, which enhanced the acetylation activity of p300 [11], we set out to investigate whether EGR1 expression levels could be elevated by MAML1. First, we transfected embryonic kidney (HEK-293) cells with *MAML1* siRNA or control (Ctrl) siRNA, and cultured the cells in the presence of TPA to induce expression of EGR1. The proteins were detected by immunoblotting with antibodies recognizing MAML1, EGR1 and GAPDH. Expression of EGR1 was induced in cells cultured with TPA (Figure 1A, lanes 3 and 4); whereas the levels of EGR1 were reduced in cells treated with *MAML1* siRNA (lane 4), compared to cells treated with control siRNA (lane 3). Next, we prepared whole cell extracts from HEK-293 cells stably expressing MAML1 and control HEK-293 cells, after inducing expression of EGR1 with TPA. The proteins were detected by immunoblotting with antibodies recognizing MAML1, EGR1 and GAPDH. As shown in Figure 1B, EGR1 was induced by TPA (lanes 2 and 4) and the expression of EGR1 significantly increased in cells overexpressing MAML1 (lane 4). Next, we investigated whether MAML1 affected *EGR1* mRNA levels. HEK-293 cells were transfected with *MAML1* siRNA or control siRNA, cultured with TPA for 2 hours, and *EGR1* mRNA expression was analyzed by real-time RT-PCR. Transfection of HEK-293 cells with *MAML1* siRNA significantly reduced the expression of *EGR1* mRNA, compared to cells transfected with control siRNA (Figure 1C). In agreement with this observation, *EGR1* mRNA increased in the MAML1-expressing cell line after induction with TPA, compared to control HEK-293 cells (Figure 1D). Thus, our data suggested that MAML1 might be involved in the regulation of EGR1 mRNA and protein expression in embryonic kidney cells.

EGR1 has a distinct spatio-temporal expression pattern during nephrogenesis [19], and disturbances in the regulation of EGR1 protein expression can lead to unwanted effects on normal cell fate. Overexpression of EGR1 has been reported to increase the proliferation of kidney cells and enhance tumorigenesis [21]. Therefore, proteins affecting the expression of EGR1, such as MAML1, might ultimately be connected to the EGR1 signaling pathway, which determines cell fate.

### MAML1 physically interacts with EGR1

**Figure 2:**
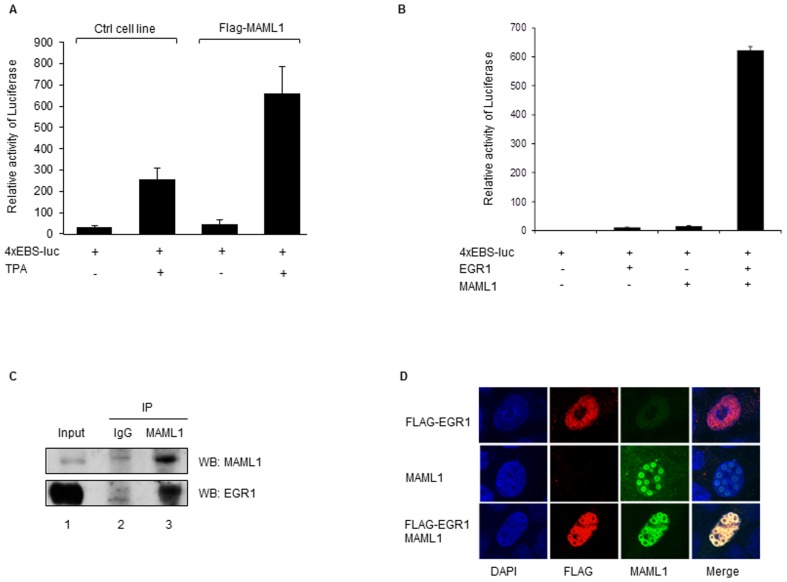
MAML1 physically and functionally interacts with EGR1. (A) FLAG-MAML1 expressing cells and HEK-293 control cells were transfected with a luciferase reporter containing four EGR1 binding sites (4xEBS-luc) and cultured with TPA to induce expression of EGR1. The data is presented as mean ± SD. (**B**) HEK-293 cells were co-transfected with 4xEBS-luc and vectors expressing EGR1 and MAML1. (**C**) Whole-cell extracts were prepared from HEK-293 cells and MAML1 protein was immunoprecipitated using an antibody recognizing MAML1. The proteins were separated by SDS-PAGE and analyzed by Western blotting using antibodies recognizing MAML1 and EGR1. (**D**) Vectors expressing FLAG-EGR1 and MAML1 were co-transfected into HCT116 cells; after 24 h the cells were immunostained using the indicated antibodies and analyzed by confocal microscopy with 100× oil lens.

We next investigated whether MAML1 could induce expression of an EGR1 target promoter, by transfecting the 4xEBS-luc reporter containing four EGR1 binding sites into cells expressing FLAG-MAML1 and control HEK-293 cells, and culturing the cells with TPA. The reporter activity increased when EGR1 was induced in control cells using TPA; however, the reporter activity was enhanced almost three fold in FLAG-MAML1 cells cultured with TPA, compared to control cells cultured with TPA (Figure 2A). In addition, HEK-293 cells were co-transfected with 4xEBS-luc and vectors expressing EGR1 and/or MAML1. While both EGR1 and MAML1 increased the activity of the EGR1 promoter; together EGR1 and MAML1 synergistically induced a 500-fold increase in the activity of the EGR1 target promoter (Figure 2B). Thus, our data suggest MAML1 may be recruited to promoters regulated by EGR1, to increase expression of the genes regulated by EGR1.

To elucidate if MAML1 interacts with EGR1, whole-cell extracts were prepared from HEK-293 cells and MAML1 was immunoprecipitated using an antibody recognizing MAML1. The proteins were separated by SDS-PAGE and Western blotting was performed using antibodies recognizing MAML1 and EGR1. As shown in Figure 2C, MAML1 and EGR1 interacted in the cellular extracts (lane 3). To determine whether EGR1 colocalized with MAML1, HCT116 cells were co-transfected with FLAG-EGR1 and MAML1 vectors and immunostained with antibodies recognizing FLAG and MAML1. EGR1 and MAML1 were strongly colocalized in the nucleus (Figure 2D). We noticed that the expression pattern of EGR1 in the nucleus changed in the presence of MAML1. We, and others, have previously reported that MAML1 colocalize MAML1-interacting proteins to nuclear bodies [24]. Our data suggest that EGR1 and MAML1 may form part of a transcription enhancer complex, which can increase the expression of genes with EGR1 binding sites. Both MAML1 and EGR1 are endogenously expressed in embryonic kidney cells, and both of these transcription factors have been shown to regulate gene expression during developmental processes [25], [26]; however, it remains to be elucidated whether MAML1 and EGR1 cooperate to regulate any of these genes.

### MAML1 and EGR1 synergistically activate the p300 promoter

**Figure 3:**
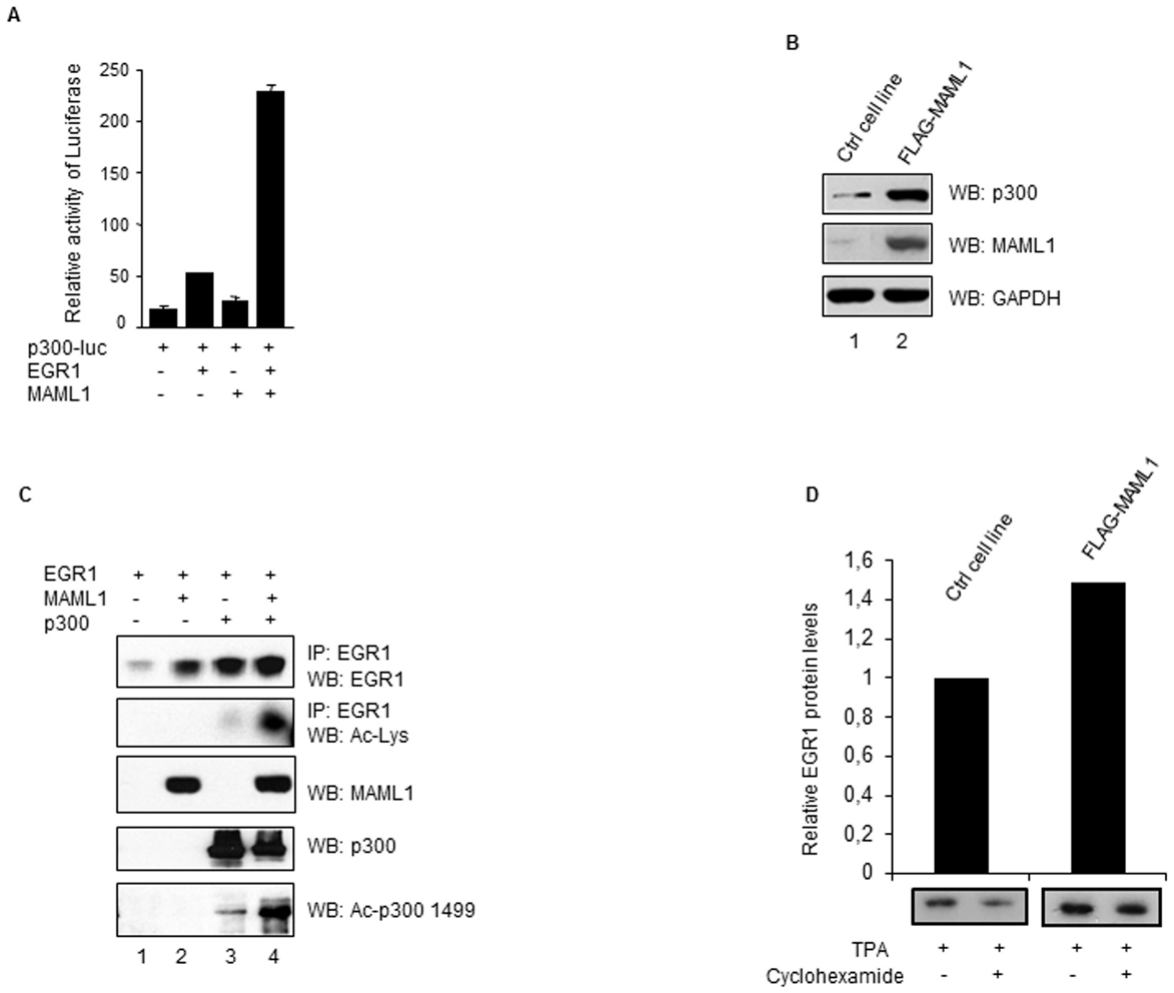
Activity of the p300 promoter is increased by MAML1. (**A**) HEK-293 cells were co-transfected with a p300-luc reporter, EGR1 and MAML1, then cultured in the presence of TPA to induce expression of EGR1. The data is presented as mean ± SD. (**B**) Whole cell extracts were prepared from FLAG-MAML1 transfected and control HEK-293 cells (Ctrl) and MAML1, p300 and GAPDH were detected by Western blotting. (**C**) Whole-cell extracts were prepared from HEK-293 cells transfected with vectors expressing FLAG-EGR, MAML1 and p300; FLAG-EGR1 was immunoprecipitated using an antibody recognizing the FLAG epitope. The proteins were separated by SDS-PAGE and detected by Western blotting using antibodies recognizing EGR1, acetylated lysines in EGR1, MAML1, p300 and acetylated lysine1499 of p300. (**D**) HEK-293 cells stably expressing FLAG-MAML1 and HEK-293 control cells were cultured with TPA (2 h) in the presence or absence of cyclohexamide (1) and EGR1 protein expression was analyzed by Western blotting using an antibody recognizing EGR1. In the graph, EGR1 protein expression in the MAML1 stable cell line is expressed relative to control cells treated with cyclohexamide. The ratio of band intensities before the addition of cycloheximide and after 1 h of treatment with cycloheximide was determined with Image J.

The p300 promoter has been reported to contain EGR1 binding sites and to be regulated by EGR1 [18]. Therefore, we co-transfected HEK-293 cells with a p300-luc reporter and vectors expressing EGR1 and MAML1, and then cultured the cells in the presence of TPA. EGR1 alone activated the p300-luc reporter in the presence of TPA, and co-transfection of MAML1 with EGR1 strongly increased the activity of the p300 reporter (Figure 3A). We hypothesized that MAML1 may regulate the expression of p300, as well as the acetylation activity of p300 [11]. Lysates were prepared from a FLAG-MAML1 cell line and HEK-293 control cells, and the levels of p300 were analyzed by Western blotting using an antibody recognizing p300. The expression of p300 increased in the MAML1-expressing cell line, compared to control cells (Figure 3B, compare lane 2); whereas the expression level of GAPDH, which is not a MAML1 target, was similar in both cell lines (lanes 1 and 2).

EGR1 is a substrate for acetylation by p300 [18], and we have previously shown that MAML1 regulates p300 activity by increasing p300 autoacetylation [11]. Therefore, we investigated whether MAML1 regulates p300-dependent acetylation of EGR1. Whole-cell extracts were prepared from HEK-293 cells and EGR1 was immunoprecipitated using an antibody recognizing EGR1. The proteins were separated by SDS-PAGE and Western blotting was performed using antibodies recognizing EGR1, the acetylated lysines in EGR1, MAML1, p300 and the acetylated lysine at 1499 of p300 (a marker of p300 autoacetylation). We noticed that expression of EGR1 increased in the presence of co-expressed MAML1 and/or p300 (Figure 3C, compare lane 1 to lanes 2–4). However, we only detected acetylated EGR1 in the presence of co-expressed p300 (lanes 3 and 4), which strongly increased in the presence of MAML1 (lane 4). The increased expression of EGR1 protein in the presence of co-expressed MAML1 (lane 2) might be due to MAML1-dependent up-regulation of EGR1 mRNA (see Figure 1). In agreement with our earlier observations, p300 autoacetylation was significantly increased by MAML1 (compare lanes 3 and 4). Thus, we presume that autoacetylated p300 acetylates EGR1 more efficiently.

Numerous reports have described how acetylation may increase protein stability [27]. Therefore, we induced expression of EGR1 using TPA in cells stably expressing MAML1 and HEK-293 control cells. After 1 h incubation with cyclohexamide, significantly more EGR1 protein was present in the MAML1-expressing cell line compared to control cells (Figure 3D). Thus, we suggest that MAML1 may be involved in regulating the stability of EGR1, possibly by increasing the acetylation of EGR1. Acetylation of EGR1 by p300 has been reported to increase the protein levels of EGR1, to stimulate expression of cell growth and survival genes such as FGF2, PDGFB, TGFB1 and IGF2, and hence plays a crucial role in prostate cancer [15], [18]. As EGR1 is expressed at high levels in prostate cancer and also certain renal cancers [21], [22], it remains to be investigated whether p300-dependent acetylation of EGR1 affects the expression of EGR1 target genes or plays a role in the development of renal cancer.

### The N-terminus of MAML1 cooperates with EGR1 during transcriptional activation

**Figure 4:**
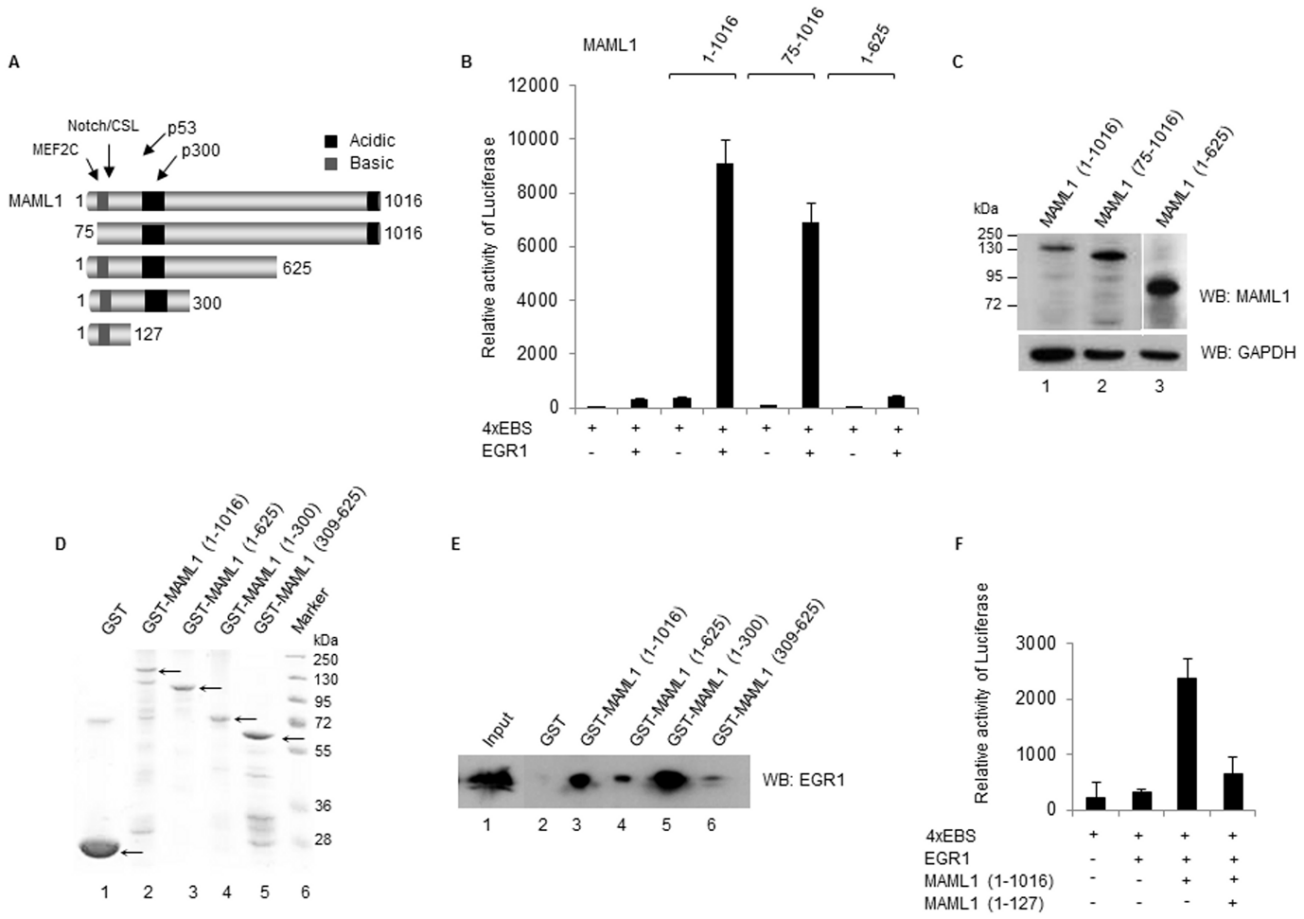
The N-terminus of MAML1 acts synergistically with EGR1 to activate transcription of EGR1-target genes. (**A**) Schematic illustration of the MAML1 domains. (**B**) HEK-293 cells were co-transfected with 4xEBS-luc and vectors expressing EGR1, MAML1 and truncations of MAML1, as indicated in the figure. The data is presented as mean ± SD. (**C**) Western blots showing the expression levels of MAML1 full-length and mutant proteins in HEK-293 cells. (**D**) PageBlue Protein-stained SDS-gel showing migration of the purified GST-tagged MAML1 proteins used for protein-protein interaction assay. (**E**) GST-tagged MAML1 derivatives and GST coupled to glutathione-Sepharose beads were incubated with HEK-293 whole cell extracts, and MAML1-interacting EGR1 was detected by immunoblotting. The input represents 2% of the whole cell extract used in the binding reaction. (**F**) HEK-293 cells were co-transfected with 4xEBS-luc and vectors expressing EGR1, MAML1 (1–1016) and (1–127). The data is presented as mean ± SD.

The MAML1 protein contains various domains, which are important for protein interactions and the function of MAML1 (see schematic in Figure 4A). Amino acids 1–74 of MAML1 interact with the Notch ICD and MEF2C, while amino acids 75–305 are important for the interaction with p300. The N-terminal domain of MAML1 also interacts with p53 and GSK3ß [24]. To investigate which MAML1 domain is required for activation of the promoters regulated by EGR1, plasmids expressing EGR1 and various MAML1 domains were co-transfected with the 4xEBS-luc reporter, which contains four EGR1 binding sites, into HEK-293 cells. As shown in Figure 4B, MAML1(1–1016) and (75–1016) strongly enhanced luciferase reporter gene activity in the presence of EGR1, while MAML1(1–625) had no detectable effect on EGR1-promoter activation. MAML1 (1–1016) and (75–1016) were expressed at similar levels in the cell culture assay, while we detected higher levels of MAML1(1–625) (Figure 4C).

In order to examine which region of MAML1 interacts with EGR1, full-length GST-tagged MAML1 protein and MAML1 derivatives (Figure 4D) were incubated with whole cell extracts from HEK293 cells transfected with EGR1. EGR1 interacted strongly with full-length GST-MAML1 protein and MAML1(1–300); but only showed a week interaction with MAML1 (1–625) and (309–625) (Figure 4E). Since we detected a strong EGR1-interaction especially with MAML1(1–300), we further investigated the role of the N-terminus MAML1 in EGR1 transcription by co-transfecting HEK-293 cells with the 4xEBS-luc reporter and plasmids expressing EGR1, MAML1 full-length and MAML1(1–127). As shown in Figure 4F, MAML1(1–127) repressed the MAML1-EGR1 synergistic activation of the EGR1-promoter. We conclude that a domain within amino acids 75–127 of MAML1 may be important for the synergistic effect of MAML1 and EGR1 on gene transcription. The MAML1 N-terminus is reported to interact with Notch, MEF2C, p53 and GSK3beta, and to play an important role in the transcriptional activity of these factors [1], [2], [5], [6], [13]. The N-terminal domain of MAML1 is also required for the interaction of MAML1 with p300, which leads to increased p300 autoacetylation and HAT activity [12]. In addition, MAML1 contains two N-terminal SUMOylation sites (K217 and K299) which repress MAML1 activity [14]. Therefore, the interactions of MAML1 should be further investigated, in order to determine whether the interaction of EGR1 with MAML1 occurs in competition, or perhaps in synergy, with other transcription factors such as Notch and p53.

### MAML1 positively correlates with EGR1 and p300 in kidney cancer

**Figure 5:**
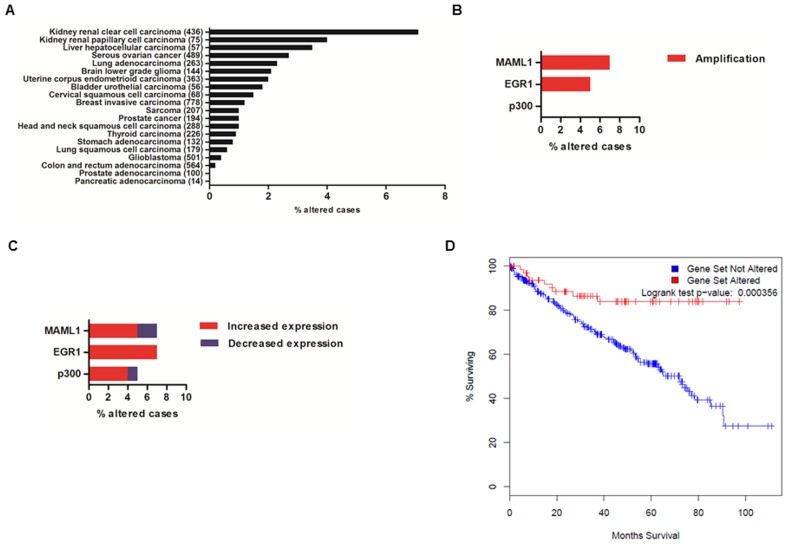
Assessment of *MAML1*, *EGR1* and *p300* expression profiles in cancer tissues using the cBio Cancer Genomics Portal. (**A**) Percentage of cancer cases with copy number alterations (x-axis), including amplification and homozygous deletions from 20 different cancer studies (y-axis) (a total of 5134 samples). The number of assessed samples for each cancer type is indicated in brackets on the y-axis (**B**) Percentage of cancer cases with copy number alterations in *MAML1*, *EGR1* and *p300* from 436 renal clear cell carcinoma samples; 31/436 cases displayed genomic amplification in one or more of the selected gene triad. (**C**) Percentage of cases with mRNA level alterations in *MAML1*, *EGR1* and *p300* from 419 renal clear cell carcinoma samples with available mRNA expression data; 69/419 cases displayed increased or decreased expression of one or more of the selected gene triad. (**D**) Kaplan-Meier plot of overall survival for 419 kidney renal clear cell carcinoma patients, based on altered mRNA expression of *MAML1*, *EGR1* and *p300*. The patients were divided into two groups based on “alterations in the gene set” (n = 69) or “no alterations in the gene set” (n = 349). One sample was removed from the initial mRNA analysis due to missing survival data information. A significant difference in overall survival was observed (*p* = 0.000356; log-rank test).

Based on the strong mechanistic evidence presented for co-regulation between MAML1, EGR1 and p300, we assessed a genomics database for such relationship. Our bioinformatic analysis of copy-number alterations (including amplifications and homozygous deletions) in the *MAML1*, *EGR1* and *p300* gene set revealed alterations in 18 of the 20 cancer studies assessed (Figure 5A). The highest level of alteration for the gene set was observed in renal clear cell carcinoma patients (7%) and renal papillary cell carcinoma patients (4%). Around 3% of the liver hepatocellular carcinoma and serous ovarian carcinoma patients displayed copy-number alterations for the gene set, while the other 14 cancer types showed alterations around 2% or below (Figure 5A). In contrast, no significant alterations were seen for pancreatic and prostate adenocarcinoma. Further analyses of the copy number changes in renal clear cell carcinoma showed that *MAML1* was amplified in 7% of the samples and *EGR1* was amplified in 5% of the samples (Figure 5B). *MAML1* and *EGR1* co-amplifications were observed in 61% of the samples from patients with either *MAML1* or *EGR1* amplification (data not shown).

Analysis of the mRNA expression levels extracted from the Cancer Genomics Portal (see Materials and Methods) indicated increased expression of *MAML1*, *EGR1* and *p300* in 5%, 7% and 4% of the renal clear cell carcinoma samples, respectively (Figure 5C). However, reduced expression of *MAML1* and *p300* were noted in 2% and 1% of the renal clear cell carcinoma samples, respectively. Expression changes in two or more genes from the *MAML1*, *EGR1* and *p300* gene set was observed in approximately 19% of the renal clear cell carcinoma samples (data not shown). The association between altered expression of the gene set at the mRNA level and overall survival in renal clear cell carcinoma was assessed. Kaplan-Meier analysis of the group of 69 patients with differential expression of at least one gene from the gene set (gene set altered) and the group of 349 patients with no alterations (gene set not altered) reveled altered gene expression had a significant impact on overall survival (Figure 5D). Alterations in the gene set were associated with increased overall survival in renal clear cell carcinoma (*p* = 0.000356) compared to the unaltered gene set. Similar analysis revealed that copy-number alterations had no significant effect on overall survival (data not shown).

In conclusion, we provide evidence that the transcriptional coactivator MAML1 acts cooperatively with EGR1 to increase transcriptional activity of the EGR1 and p300 promoters in embryonic kidney cells. Moreover, using public data sets we have determined that alterations in the *MAML1*, *EGR1* and *p300* gene set were associated with increased overall survival in renal clear cell carcinoma. The genes encoding *EGR1* (5q31.1) and *CSF1R* (5q33–q35) have been reported as the most frequently altered genes on chromosome 5 in renal cell clear carcinoma [22]. As the *MAML1* gene is located at 5q35.3, in close proximity to EGR1 and CSFIR, we speculate that the *MAML1* gene may be altered by similar or the same mechanisms as *EGR1* and *CSF1R*. Further experiments will focus on elucidating whether the gene loci within 5q31–q35 are targets of altered epigenetic regulation in renal cell clear carcinoma, which may result in altered expression of these genes. Future in vivo studies will also be required to verify the importance of the MAML1-EGR1 interaction in different cellular contexts, and determine whether MAML1-dependent acetylation of EGR1 play a role in tumorigenesis.
