# Expression of Concern: MAML1 Acts Cooperatively with EGR1 to Activate EGR1-Regulated Promoters: Implications for Nephrogenesis and the Development of Renal Cancer

**DOI:** 10.1371/journal.pone.0344927

**Published:** 2026-03-16

**Authors:** The PLOS One Editors

After publication of this article [1], concerns were raised about Figs 1- 4. Specifically, when color levels are adjusted, there appear to be vertical discontinuities between the following lanes:

- Lanes 2 and 3 of the WB: EGR1 panel in Fig 1A.
- Lanes 2 and 3 of the WB: GAPDH panel in Fig 1A.
- Lanes 1 and 2 of the WB: EGR1 panel in Fig 2C.
- Lanes 1 and 2 of the WB: MAML1 panel in Fig 3C
- Lanes 1 and 2 in Fig 4E.

Additionally, in Fig 4C, the disclosed splice line between MAML1 (75–1016) and MAML1 (1–625) of the WB: MAML1 panel is not present in the corresponding WB: GAPDH panel.

The first author agreed with the concerns noted in Fig 4C, but not the concerns noted in the other figures. Authors MLH and BF further stated that the concerns outlined above have no impact on the scientific conclusions of the study.

Authors MLH and BF stated that the original images underlying the figures presented in [1] are no longer available as the institutional data retention period has elapsed. In the absence of the underlying image data, the above concerns cannot be fully resolved.

The *PLOS One* Editors issue this Expression of Concern to notify readers of the above concerns.

The corresponding author of [1] is deceased. Henceforth, correspondence may be sent to the first author, MLH (magnushassno@gmail.com).
